# Creating a Pilot Educational Psychiatry Website: Opportunities, Barriers, and Next Steps

**DOI:** 10.2196/mededu.4580

**Published:** 2015-11-05

**Authors:** John Torous, Ryan O'Connor, Jamie Franzen, Caitlin Snow, Robert Boland, Robert Kitts

**Affiliations:** ^1^Department of PsychiatryBrigham and Women's HospitalHarvard Medical SchoolBoston, MAUnited States; ^2^New York-Presbyterian HospitalDepartment of PsychiatryWeill Cornell Medical CollegeNew York City, NYUnited States; ^3^Department of PsychiatryBrigham and Women\'s HospitalHarvard Medical SchoolBoston, MAUnited States; ^4^Boston Children’s HospitalDepartment of PsychiatryHarvard Medical SchoolBoston, MAUnited States

**Keywords:** Psychiatry, Internet, Online, Education, Website

## Abstract

**Background:**

While medical students and residents may be utilizing websites as online learning resources, medical trainees and educators now have the opportunity to create such educational websites and digital tools on their own. However, the process and theory of building educational websites for medical education have not yet been fully explored.

**Objective:**

To understand the opportunities, barriers, and process of creating a novel medical educational website.

**Methods:**

We created a pilot psychiatric educational website to better understand the options, opportunities, challenges, and processes involved in the creation of a psychiatric educational website. We sought to integrate visual and interactive Web design elements to underscore the potential of such Web technology.

**Results:**

A pilot website (PsychOnCall) was created to demonstrate the potential of Web technology in medical and psychiatric education.

**Conclusions:**

Creating an educational website is now technically easier than ever before, and the primary challenge no longer is technology but rather the creation, validation, and maintenance of information for such websites as well as translating text-based didactics into visual and interactive tools. Medical educators can influence the design and implementation of online educational resources through creating their own websites and engaging medical students and residents in the process.

## Introduction

In the dynamic and rapidly advancing modern technological landscape, the role of digital learning tools in medical education is an emerging area of study. Although much has been written about online resources and learning in medical education, there is a lack of studies on physician’s role in the development of these digital tools. This paper describes the creation of a pilot psychiatric educational website, and relevant considerations about the present and future role of physician-developed Web technology in medical education.

Medical students and residents are increasingly relying on Web technologies as their primary educational resources. A recent survey of fourth-year medical students found that on a daily basis, for educational purposes, 76% used Google searches, 83% used Wikipedia, 57% used UpToDate, and only 8% used bibliographic databases. [1]. Another survey study of residents of psychiatry revealed that the majority of respondents relied on online resources instead of textbooks, with the most utilized website being UpToDate, closely followed by Wikipedia and PubMed [2].

The variables driving the preference for and selection of online educational resources are multifold. Although there are numerous novel and effective uses of technology in medical education including gamification [3], feedback dashboards [4], social media [5], and virtual reality [6], websites remain the most frequently utilized digital resource. The high rate of use of Wikipedia and similar resources suggests that medical trainees are relying in part on user-created content for their education. Furthermore, in a recent survey of pediatric residents about preferences for educational website functions, 100% of respondents wanted pictures, 93.2% interactivity, 88.9% digital videos, 88.6% links to articles and research, and 86.4% graphics and animation [7]. These reports suggest that medical trainees value the interactive and multimedia potential of Web-based educational resources for learning and understanding their subject materials.

Modern Web technology now offers a novel opportunity for medical educators and trainees to not only be consumers of educational websites, but also participate in their creation. Technology has evolved to make the development of custom websites affordable, feasible, and educationally valuable. Understanding the process of developing digital technologies for medical and psychiatric education provides an opportunity for physician educators to leverage technology to better engage learners around relevant and quality content. A firsthand narrative describing the construction of a novel educational website for psychiatry trainees, PsychOnCall, provides a lens through which one can understand the current barriers, capabilities, and future opportunities for physicians in integrating technology development and teaching.

The value of understanding the creation and development of websites is best understood and framed in the context of advances in medical education theory. Adult learning theory, which highlights the active role of the learners’ aspirations, as applied to residents of psychiatry suggests they are motivated more by personal interests and clinical relevance of topics than by training requirements [8]. The flipped classroom model, in which residents learn foundational material on their own and then use didactic time to discuss or apply such knowledge, encourages active learning and self-discovery of knowledge [9]. In the same vein, while a website may classically be seen as source of knowledge for learning, in the flipped classroom model it can also serve as a source of knowledge through creating content. Using Web technology, collaboration and active learning are no longer limited to just physical space, and several successful educational websites, such as that of the Kahn Academy, have been developed to realize this potential. However, there currently does not exist any widely available psychiatry-specific resource.

When it comes to training faculties and trainees, the competency of technologically minded individuals varies significantly, just as the case with wide range in competency in clinical knowledge and research literacy among these individuals. The creation and maintenance of a Web-based educational tool with clinically sound educational material can potentially serve as a venue to merge bidirectional teaching and learning in all 3 realms, namely, technology, clinical knowledge, and research literacy. More technologically minded trainees could educate and engage faculty members on the use and benefits of technology through this endeavor, while faculty members can educate and engage the trainees in systematically reviewing the quality of clinical material to be used for the website. The latter thereby reinforcing the cliché “Do not believe everything you read on the Internet.” It could also foster discussions on ways in which different learners learn and how to optimize the use of technology to benefit multiple learning styles. This endeavor may also help identify trainees and faculties who may benefit from or be interested in further training in biomedical and clinical informatics, which is a growing field in medicine.

Thus, it seems that advances in technology and educational theory offer the opportunity for medical trainees and training programs to play an active role in the creation of an educational website that is visually appealing, dynamic, and interactive, where both teachers and trainees can learn from each other. Our goal was not to build that website but to rather explore the process to provide a pilot experience for an eventual large-scale project recognizing the value in understanding the development of educational technologies [10]. While we discuss the process of constructing our single specific website, PsychOnCall, we believe that the process of creating this website could provide valuable generalized lessons regarding both the opportunities and barriers that any resident, clerkship director, or program director will likely face when trying to build their own educational website.

## Methods

### PsychOnCall: A Pilot Psychiatric Educational Website

A pilot psychiatric educational website (PsychOnCall) was created to better understand the options, opportunities, challenges, and processes involved in the creation of a psychiatric educational website. We sought to integrate visual and interactive Web design elements to underscore the potential of such Web technology. These elements are described in the following sections.

### Thinking Visual

Web technology has advanced to a point where the barriers toward creating a visual, interactive, and dynamic website have been greatly reduced. Code to create such graphics is freely available at websites such as D3. However, the challenge now lies not in the coding, but rather in translating classically abstract and static psychiatric concepts into visual and dynamic templates.

**Figure 1:**
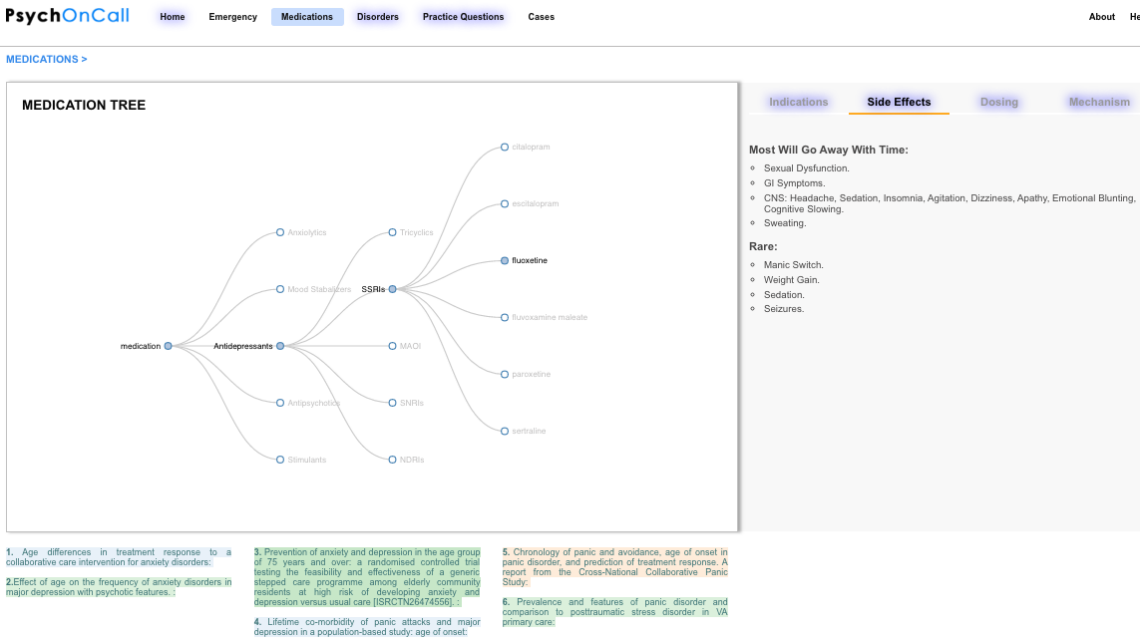
A dynamic medication tree can offer a ‘big picture’ view of how a medication fits into a class/family while simultaneously presetting specific information for that medication.

For example, in planning how to present medications we did not want to just provide a list of medications but rather create an interactive branching tree that responds to users clicking on various levels of the tree (Figure 1). This dynamic approach allows the user to visualize the classification and hierarchy of a specific medication while still providing detailed information as well as making alternative choices apparent. A further advantage is the ability to provide customized links to the primary source literature, which can change with each click so that, for example, core papers on fluoxetine can be displayed when the user selects such a link (Figure 1).

**Figure 2:**
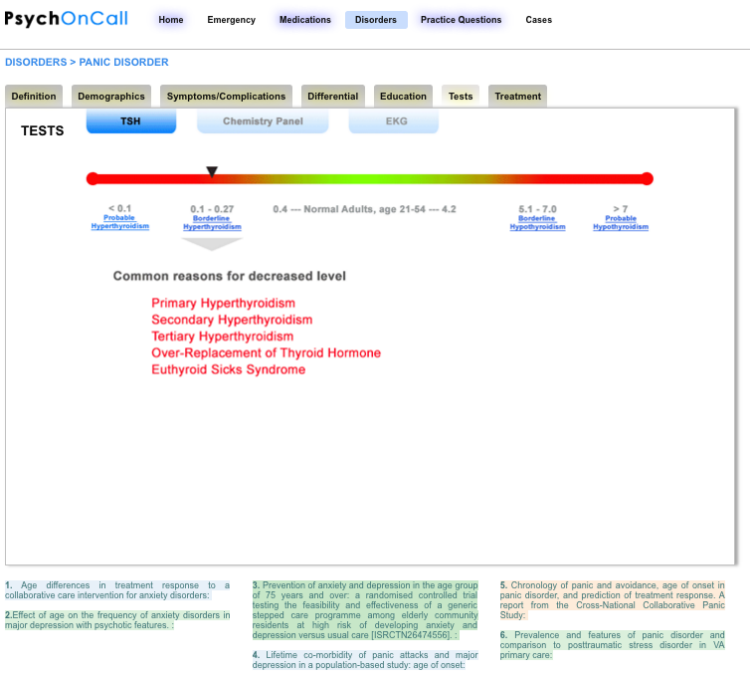
Dynamic slider bars and other interactive features can present information on lab tests in a more engaging manner as seen above for a thyroid lab test.

Another example of transforming more static data into a dynamic learning experience is seen in the presentation of lab value. Classically, lab reference data may be displayed on a printed chart; however, by using Web technology these data can be represented with a slider bar (Figure 2) that displays relevant differential diagnosis information depending on where the user moves the slider bar.

**Figure 3:**
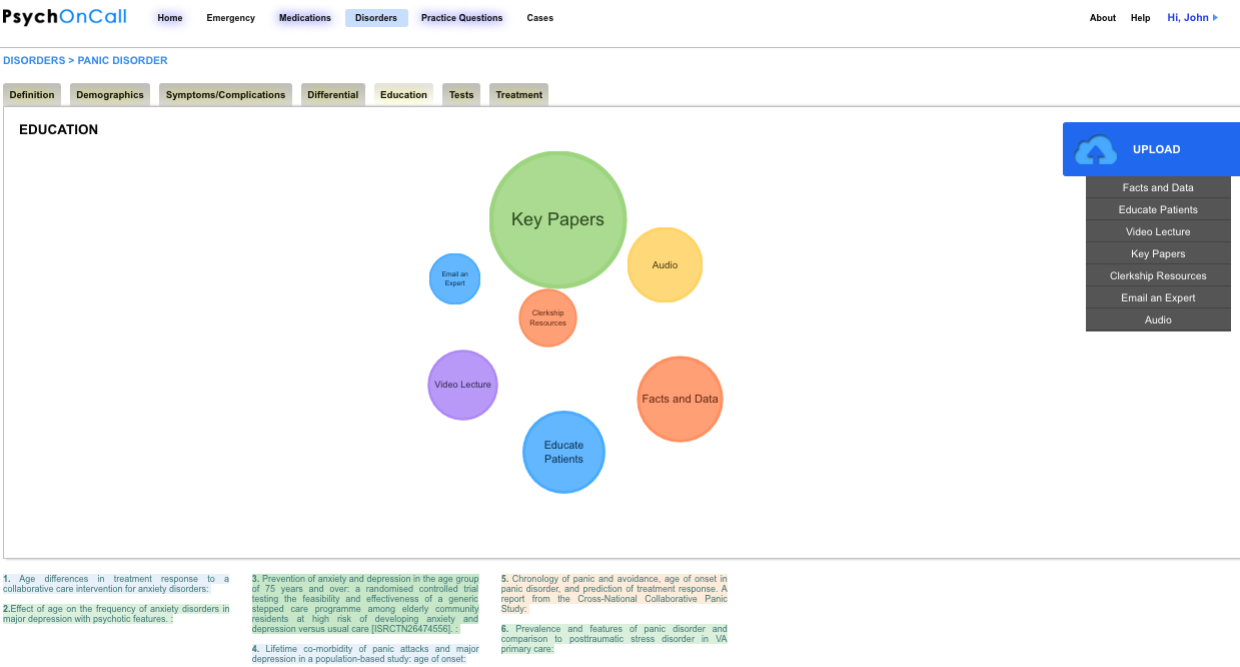
Web technology can make learning more social and offers the opportunity for trainees to contribute their own ideas and links in the form of papers, videos, audio, lectures, and more as seen above.

In addition, such a website can also be used for collaboration and information sharing between trainees. We created a page (Figure 3) designed for sharing video lectures, educational material, core papers, podcasts, and important websites. As trainees could easily upload their own resources, such a website has the potential to foster digital collaboration and empower residents to create and disseminate their own teaching materials.

**Figure 4:**
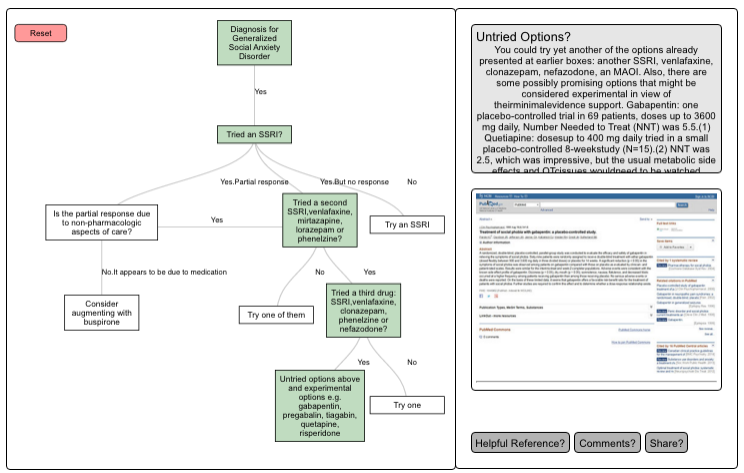
Algorithms can be programmed to be interactive and display relevant links to PubMed papers. This algorithm is derived from the social anxiety algorithm created by the psychopharmacology algorithm project at the Harvard South Shore Program.

Finally, the interactivity of websites is well suited to make treatment algorithms more engaging, dynamic, and educational. We collaborated with the psychopharmacology algorithm project at the Harvard South Shore Psychiatry to use one of their algorithms to pilot on our website (Figure 4). The ability to directly visualize the PubMed links related to each step in the algorithm may remind trainees to consider the evidence and literature that individual recommendations are based on, and to learn more by exploring individual papers.

The possibilities are broad and advances in Web technology will continue to offer new tools to visualize and interact with educational information. We do not claim to know the optimal way to display psychiatric material in a visual and dynamic manner but hope this will become an active area of study by educational experts.

## Results

### Overview

The pilot website (PsychOnCall) created demonstrated the potential of Web technology in medical and psychiatric education. With the created website, the opportunities, barriers, and process of creating a novel medical educational website were evaluated.

### Challenges

Even given the pilot nature of our project and goal to explore the potential of Web technology for creating a psychiatric educational website rather than building a fully usable website, we ran into the following challenges, which would likely have to be well thought out before beginning a large-scale website project.

### Cost

Although there are many approaches toward building a website, many of them will involve some direct cost. Utilizing template website services such as Weebly, WordPress, or Squarespace, it possible to both create and host a professional appearing website at low cost. Custom programming will cost more, although many academic departments or universities often have in-house programmers that may reduce cost. Finally, tech-savvy residents and educators may actually be able to accomplish the majority of required work. While cost is always an important issue, it is worth noting that 16 years ago, computer technology in a psychiatry residency was viewed as an investment [11], but today it is seen as a necessity.

### Content

Finding a source of high-quality and accurate content related to psychiatry education can prove difficult as residents do not have the experience or credentials to create one alone. Active review and vetting by senior faculty members are necessary to ensure any uploaded information is accurate. Thus, before creating an educational website, it will be necessary to ensure good collaboration between faculty and residents with an agreed upon content review process.

### Updating

As the knowledge base of psychiatry keeps rapidly expanding, it will be necessary to ensure that any information posted on an educational website is well curated and kept up-to-date. As the website expands in content, this updating process may become substantial and require dedicated efforts. Thus, before building a website, it is necessary to have a long-term framework for its continuation. While individual residents may graduate or faculty may leave, a plan should be in place to ensure that the website will remain updated and accurate in the long term.

### Access

While an educational website offers the potential to easily share information with anyone in the world, understanding both who should and who can access the website is important. The risk of the public accessing the website and creation of an unintended patient-doctor relationship must be kept in mind and any website publicly posted should be well reviewed by the legal team of the hospital concerned and contain all necessary local, state, and federal disclosures. Password protecting the website, thereby limiting access to residents or medical students, is a potential solution. Fully understanding the desired accessibility before building the website would be important in shaping both its security and scope.

## Discussion

While building our pilot website we focused on exploring the potentials of Web technology to enhance general medical as well as psychiatric education and the understanding of the process necessary to construct such a website. Online educational websites remain a nascent field and there are many exciting avenues for such to develop along. Although there are technological barriers to implementation, we believe that these barriers will continue to diminish and that the true barrier at this point is the design, translation, and validation of core psychiatric concepts presented on the page and their conversion into interactive and visual formats.

Rather than be flooded by a sea of websites, medical as well as psychiatric educators now have the opportunity to develop their own online resources as a hub of education. While the creation of websites is not novel, the increased reliance on them by trainees and the increased ease of creating them is novel and important. Using Web technology, now at a point where trainees can create websites as in our example, technology is no longer only a medium for educational knowledge but also a tool to be planned, developed, and learned.

While the potentials of digital learning to provide personalized adaptive or collaborative learning have been widely discussed and defined [12], actually implementing such in clinical education has been less well documented. Considering technology itself as a tool to be learned by medical trainees has been even less discussed. As we demonstrated, the actual creation of such a website even with a social/collaborative component is technically feasible and easily constructed. The challenge now is observing and understanding how trainees may actually use such a website and how various learning theories translate into an online reality. Medical and psychiatric educators now have the opportunity to conduct such studies and gather actual user data.

However, in order for such to succeed there should be several well-established processes before construction of any actual website. Understanding how content will be created and who will review and approve it are barriers that will likely become of increasing importance. Likewise, understanding how such content will be updated is another important consideration. A potential solution grounded in adult learning theory and the open classroom model is that trainees will provide the content and actively review the quality of the material with faculty as a shared process, which would simultaneously promote education in research literacy. However, the actual implementation of such remains unproven.

Despite the increasing prevalence of website use in medical education and psychiatry, much also remains unknown and unproven. While our paper does not present any user or subject data, our goal here is to not the highlight the merits or faults of any one unique website but rather consider the motivations and processes around creating such tools. Although there continues to be newer technologies, for example, virtual reality headsets and smart watches, websites currently remain the core technology that medical trainees continue to rely on. A better understanding of their use and the process of their creation is the important first step for medical education to better understand and utilize technology for teaching.

Although creating such processes will not be a simple task, starting to think of solutions now is important in ensuring that psychiatric educators will be able to influence the design and implementation of online educational resources. Following the recommendations of the Working Group on the 2011 conference “A 2020 Vision of Faculty Development Across the Medical Education Continuum,” we agree that technology should be used to supplement but not replace face-to-face experience, that medical schools should allocate a variety of resources and support, and that national organizations should provide funding and leadership [13]. While it is impossible to define that tipping point where the investment and necessary resources decrease and the potential educational benefit increases, perhaps psychiatric education is now at that very tipping point with the future of online educational resources in motion. At one point in time, 16 years ago to be exact, it was noted that “software [for residency administration] will require both a financial and time investment before there can be dividends” [11] and today we would argue that online educational websites today are at that same crossroads.
